# *Piscirickettsia salmonis* Cryptic Plasmids: Source of Mobile DNA and Virulence Factors

**DOI:** 10.3390/pathogens8040269

**Published:** 2019-11-28

**Authors:** Javiera Ortiz-Severín, Dante Travisany, Alejandro Maass, Francisco P. Chávez, Verónica Cambiazo

**Affiliations:** 1 Laboratorio de Bioinformática y Expresión Génica, Instituto de Nutrición y Tecnología de los Alimentos (INTA), Universidad de Chile, Santiago 7830490, Chile; javiera.o.s@gmail.com; 2 Laboratorio de Microbiología de Sistemas, Departamento de Biología, Facultad de Ciencias, Universidad de Chile, Santiago 7800003, Chile; fpchavez@uchile.cl; 3 Centro de Modelamiento Matemático (AFB170001) and Departamento de Ingeniería Matemática, Facultad de Ciencias Físicas y Matemáticas, Universidad de Chile and UMI-CNRS 2807, Santiago 8370415, Chile; dtravisany@dim.uchile.cl (D.T.); amaass@dim.uchile.cl (A.M.); 4 Fondap Center for Genome Regulation (Fondap 15090007), Universidad de Chile, Santiago 8370415, Chile

**Keywords:** fish pathogen, *Piscirickettsia salmonis*, plasmids, MGE, virulence factors, prophages, gene annotation

## Abstract

Four large cryptic plasmids were identified in the salmon pathogen *Piscirickettsia salmonis* reference strain LF-89. These plasmids appeared highly novel, with less than 7% nucleotidic identity to the nr plasmid database. Plasmid copy number analysis revealed that they are harbored in chromosome equivalent ratios. In addition to plasmid-related genes (plasmidial autonomous replication, partitioning, maintenance, and mobilization genes), mobile genetic elements such as transposases, integrases, and prophage sequences were also identified in *P. salmonis* plasmids. However, bacterial lysis was not observed upon the induction of prophages. A total of twelve putative virulence factors (VFs) were identified, in addition to two global transcriptional regulators, the widely conserved CsrA protein and the regulator Crp/Fnr. Eleven of the putative VFs were overexpressed during infection in two salmon-derived cellular infection models, supporting their role as VFs. The ubiquity of these plasmids was also confirmed by sequence similarity in the genomes of other *P. salmonis* strains. The ontology of *P. salmonis* plasmids suggests a role in bacterial fitness and adaptation to the environment as they encode proteins related to mobilization, nutrient transport and utilization, and bacterial virulence. Further functional characterization of *P. salmonis* plasmids may improve our knowledge regarding virulence and mobile elements in this intracellular pathogen.

## 1. Introduction

*Piscirickettsia salmonis* is the etiological agent of piscirickettsiosis, a disease that affects various farmed salmonids worldwide [1]. Early reports of the disease date back to 1981, but it was not until 1989—when a massive outbreak killed over 90% of Coho salmons (*Oncorhynchus kysutch*) reared in saltwater net pens near Puerto Montt, Chile—that the causative agent of the disease was identified [2]. The sick fish presented erratic swimming behavior, lethargy, inappetence, dark color and pale gills, while internal organs such as the kidney, liver, spleen, and brain were affected [1,2]. A Gram-negative, rickettsia-like bacterium isolated from infected fish was found to cause the disease in Coho salmon, and was frequently observed within cytoplasmic vacuoles inside the host cells [3]. Subsequent genetic studies analyzed the 16S rDNA sequence and classified *P. salmonis* in the γ-proteobacteria class, with phylogenetic proximity to other intracellular bacteria such as *Coxiella*, *Francisella*, and *Wolbachia* species, and it proposed to be a unique species of the new genera *Piscirickettsia* [4]. Since its isolation in 1989, strain LF-89 (ATCC VR-1361) has been the reference-type strain and its genome was sequenced and annotated, revealing a 3.2 Mb chromosome and three medium- to large-sized plasmids [5]. Later, a fourth LF-89 plasmid was identified, which was also deposited in the NCBI database (accession number NZ_CP013669.1). In January 2017, eighteen new *P. salmonis* strains were sequenced and annotated, and based on genomic comparisons [6], these 18 strains were split into two separate genogroups named after the reference strains, LF-89 or EM-90, as LF-89-like or EM-90-like. All of these strains harbor at least three plasmids each.

Plasmids are extrachromosomal circular or lineal double-stranded DNA molecules that vary in size from a few kilobases to over a hundred [7,8,9]. Plasmids are capable of autonomous replication and can be mobilized into other related bacteria [10,11,12]. Bacterial plasmids play a key role in adaptation to environmental conditions. Important biological functions are associated with plasmids in both pathogenic and non-pathogenic bacteria. For instance, in *Salmonella* (and other enteric pathogens), plasmids are responsible for phenotypic traits including virulence, resistance to heavy metals, antibiotics, and utilization of carbon sources [13,14,15,16]. Environmental bacteria also harbor plasmids that participate in niche adaptation, conferring heavy-metal [17] and antibiotic resistance [18,19], nutrient transport and degradation [20,21], and even hormone synthesis that mediates trans-kingdom interactions [20]. However, some bacteria contain plasmids with no obvious or known functions that are classified as cryptic. Cryptic plasmids are found in many naturally occurring bacteria and require further functional characterization of their coding sequences in order to elucidate their contribution to the bacterial cell cycle and pathogenesis [22,23,24,25,26].

Facultative intracellular bacteria, such as *P. salmonis*, can replicate both inside a host and extracellularly. These pathogens have variable-length genomes, with a proportion of mobile genetic elements (MGE) similar to free-living pathogens [27]. In other marine bacteria, including bacterial pathogens, an important aspect of its survival and adaptation is related to the ability to transfer information via MGE, such as phages, insertion sequences and transposons, integrons, integrative conjugative elements (ICEs), and plasmids [28,29,30]. Due to numerous environmental metagenome sequencing projects, including plasmidome and virome information, it is suggested that plasmids are the primary mobile element that transfer genetic information between bacterial genomes [31]. An estimate of 24–52% of marine bacteria contain one or more plasmids [30], related to the acquisition of advantageous traits such as nutrient intake and degradation, metal and antibiotic resistance, and virulence, among others. However, MGEs and their influence in the pathogenesis of *P. salmonis* remain to be elucidated.

The facultative intracellular nature of *P. salmonis* [32] allows it to interact both with the surrounding marine environment as a free living bacteria, and with its host as an intracellular pathogen. The presence of extrachromosomal elements in all *P. salmonis* strains sequenced to date suggests that they provide accessory traits that might be beneficial under particular conditions. Nonetheless, with the exception of the multidrug resistance plasmid p3PS10 harbored by the LF89-like AY3800B strain [33], *P. salmonis* plasmid contents and their functions have not been examined yet. In addition, a proteomic study found putative plasmid-encoded toxins in *P. salmonis* extracellular vesicles [34], which suggested a possible role in bacterial virulence of this important MGE. Considering that plasmids are highly mobile genetic elements that can be important for spreading virulence and multidrug resistance traits [13,16,35,36], here, we report the overall plasmid content of the *P. salmonis* LF-89 strain. The genetic structures of the plasmids, as well as the diversity of genes, their function and their ubiquity in the genomes of other *P. salmonis* strains were analyzed. In addition, we developed a molecular tool for the specific identification of *P. salmonis* plasmids under standard laboratory conditions. To gain further insights into the role of *P. salmonis* plasmids during host infection, we carried out in vitro infections using the salmon-derived cell lines CHSE-214 and SHK-1, and evaluated the gene expression of the annotated *P. salmonis* plasmid virulence factors (VFs). The results indicated that in comparison to exponentially growing bacterial cells, infection significantly increased the expression of 11 plasmid-encoded VFs, supporting the idea of an active role of these MGE during the pathogenesis of *P. salmonis*.

## 2. Results

### 2.1. Identification and Visualization of Extrachromosomal Elements in P. Salmonis LF-89

The first completed genome sequence of *P. salmonis* became available in 2015. Before that, eight sequencing projects deposited in the NCBI database were incomplete genomes with a number of contigs ranging from 227 to 543. Half of the projects belong to the reference strain LF-89 (ATCC VR-1361), and the other half, to field isolates. This DNA sequence configuration did not allow a precise prediction of extrachromosomal elements, such as plasmids, in *P. salmonis*. With the publication of the complete and annotated genome of the LF-89 strain, three auto-circularizing contigs where reported as predicted plasmids. Later, four plasmids were reported in the PM32597B1 and PM15972A1 strains, in the NCBI database. To corroborate these predictions, a plasmid-enriched fraction from a DNA purification obtained from the *P. salmonis* LF-89 strain was sequenced with a short-sequence/ high-precision method (Illumina GAIIx). The final assembled Illumina sequences generated four circular extrachromosomal elements of 180 kb (pPSLF89-1, accession number NZ_CP011850.1), 33.5 kb (pPSLF89-2, accession number NZ_CP011851.1), 51.2 kb (pPSLF89-3, accession number NZ_CP011852.1), and 57.4 kb (pPSLF89-4, accession number NZ_CP013669.1).

The results of the sequenced plasmids are shown in Table 1. These plasmids have a GC content similar to the chromosome (39.7% for the chromosome, 38.9% for pPSLF89-1, 40.6% for pPSLF89-2, 39.1% for pPSLF89-3, and 37.3% for pPSLF89-4), and the four plasmids differ in terms of the size and number of coding sequences (CDS). Pseudogenes comprise between 7% and 18% of plasmids genes. An average of 12.8% of all *P. salmonis* genes were found to be pseudogenes, and plasmids pPSLF89-1 and pPSLF89-2 contain an even greater percent of pseudogenes. One possible explanation for the high percent of pseudogenes in these plasmids is the amount of viral-related proteins found in them. Over 70% of pPSLF89-1 and pPSLF89-2 proteins are phage-related proteins, while only 43% and 25% of pPSF89-3- and pPSLF89-4- encoded proteins were annotated as phage-related proteins. On the other hand, about 47% of pPSLF89-1 proteins were annotated as transposons or integrases. In addition, almost 75% of the pPSLF89-1 pseudogenes are partial sequences of transposases. The BLAST online alignment tool was used to determine regions of nucleotidic similarity of *P. salmonis* complete plasmid sequences and the nr database maintained by the National Center for Biotechnology Information (NCBI plasmid database). The four *P. salmonis* plasmid sequences did not show any significant similarities to any other non-Piscirickettsial plasmids in the database, as shown ≤7% identity over the entire sequences.

**Figure 1:**
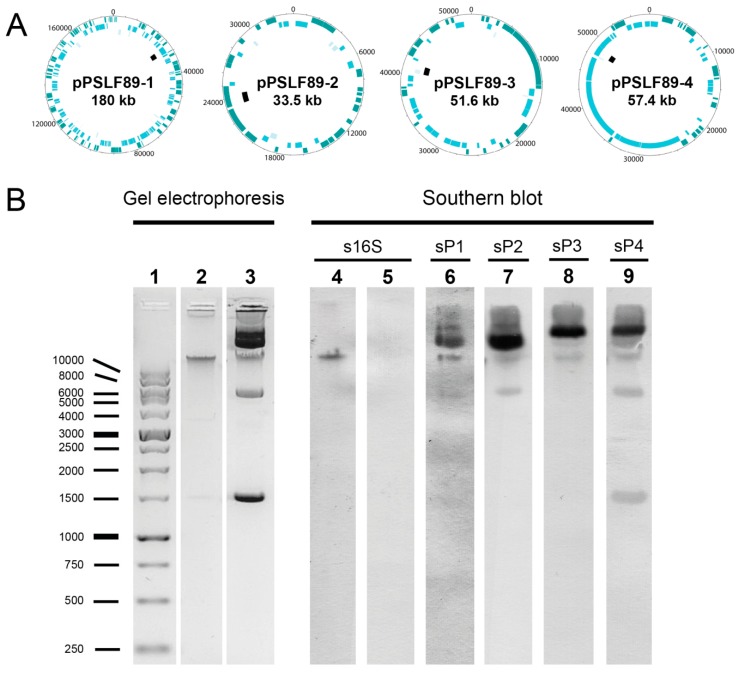
*P. salmonis* LF-89 plasmid visualization by DNA gel electrophoresis and Southern blot analysis. (**A**) Graphic representation of *P. salmonis* LF-89 plasmids and their probes. From the outside circle: in dark blue, coding sequences in sense-strand; cyan, coding sequences in antisense-strand; light blue, pseudogenes; black, Dig-labeled probe; plasmid name and its molecular weight are given. (**B**) DNA gel electrophoresis (lanes 1, 2, and 3) and Southern blot analysis with Dig-labeled probes (lanes 4–9). In the gel electrophoresis, the 1 kb DNA ladder (lane 1), the *P. salmonis* genomic DNA (lane 2) and plasmid DNA (lane 3) are shown. In the Southern blot, the genomic DNA hybridized with the 16S probe is shown in lane 4, and the plasmid DNA with the 16S probe in lane 5. Lanes 6–9 correspond to plasmid DNA hybridized with sP1 probe for pPSLF89-1 (lane 6), sP2 probe for pPSLF89-2 (lane 7), sP3 probe for pPSLF89-3 (lane 8), and sP4 probe for pPSLF89-4 (lane 9).

Unique plasmid DNA sequences comprising 1–3 kb were used to design plasmid-specific Dig-labeled probes in order to visualize the predicted plasmids of the LF-89 strain (Figure 1). As shown in Figure 1, several bands with different migration profiles were visualized after the DNA gel electrophoresis, which correlates with the prediction of the existence of plasmids in the LF-89 strain. The Southern blot successfully identified different migration patterns between plasmids, which could be useful to identify the plasmids with specific probes. Although superposition could be observed within bands when using different probes, the intensity and migration pattern of the bands differed between them. The pPSLF89-4 plasmid showed a distinct migration pattern and was easily identified as the sP4 probe marked a band near the 1.5 kb ladder.

**Figure 2:**
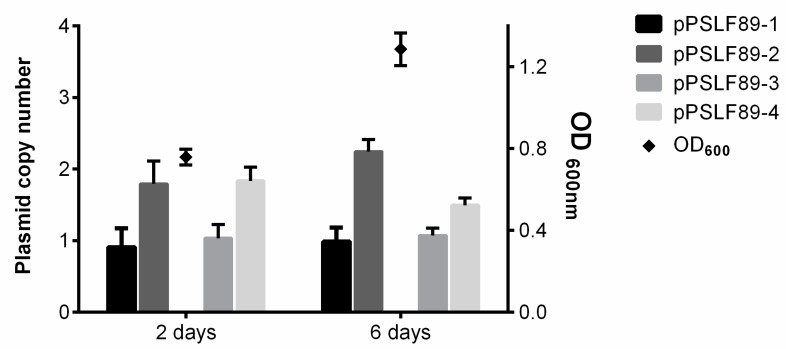
*P. salmonis* LF-89 plasmid copy number. Bacterial growth is shown as optical density measurements at 600 nm (OD_600_, right y-axis) and indicated as black diamonds with its correspondent standard deviation. The plasmid copy number was calculated by qPCR and is shown as chromosome equivalents (bars, left y-axis). The mean ± standard deviation of four independent replicates is shown.

Plasmid copy number was determined for each *P. salmonis* LF-89 plasmid using previously described methods [37]. Samples were taken from *P. salmonis* batch cultures at exponential and stationary growth phases. qPCR-based calculations revealed that all four plasmids showed low copy numbers (1–2 per cell) and were present in chromosome equivalent ratios independently of the bacterial growth phase (Figure 2). Statistical analysis showed no difference between the plasmids copy number at 2- or 6 -days (2-way ANOVA, *p* = 0.4536).

Thus, here, we reported a feasible experimental approach for visualization and copy number determination of *P. salmonis* LF-89 plasmids based on specific DNA sequences.

### 2.2. Categorization of Plasmidial Proteins in COGs

**Figure 3:**
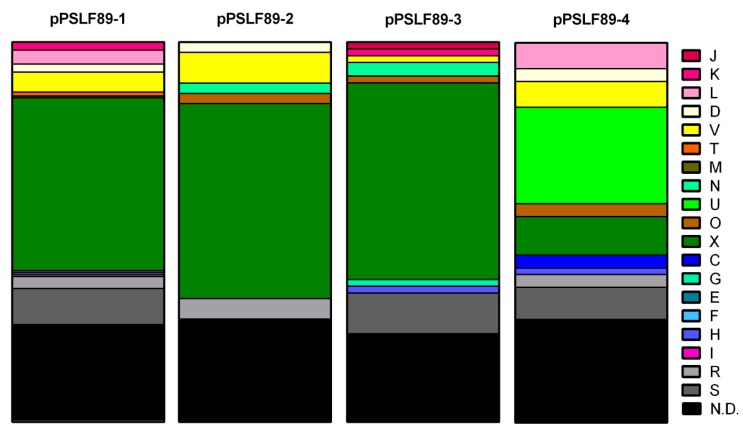
Representation of *P. salmonis* plasmid proteins categorized according to the Cluster of Orthologous Groups (COGs) database. The bar graph represents the percent of plasmid proteins categorized in each COG based on the protein sequence, related to total number of plasmid proteins, for each *P. salmonis* plasmid. The COGs found are shown as general category letter associations. (J) Translation, ribosomal structure and biogenesis; (K) transcription; (L) replication, recombination and repair; (D) cell cycle control, cell division, chromosome partitioning; (V) defense mechanisms; (T) signal transduction mechanisms; (M) cell wall/membrane/envelope biogenesis; (N) cell motility; (U) intracellular trafficking, secretion, and vesicular transport; (O) post-translational modification, protein turnover, and chaperones; (X) mobilome, prophages, transposons; (C) energy production and conversion; (G) carbohydrate transport and metabolism; (E) amino acid transport and metabolism; (F) nucleotide transport and metabolism; (H) coenzyme transport and metabolism; (I) lipid transport and metabolism; (R) general function prediction only; (S) function unknown; (N.D.) not determined.

In order to predict the functionality of these novel *P. salmonis* plasmids, we observed their gene content in more detail. For each plasmid, the Cluster of Orthologous Groups (COGs) were assigned to the predicted CDS and are shown in Figure 3 as a percent of the total predicted proteins in each plasmid (Supplementary Table S1). A high percent of plasmid proteins (25.5%) were novel and did not share common features with other proteins in genomic databases, so they could not be categorized (N.D. category, black). A large proportion of proteins with unknown function (R) or only general predicted function (S) were also found (gray and light gray). This indicates that most of *P. salmonis* plasmidial proteins and their functions have not been described before.

The most common COG is related to mobilome, prophage, and transposons (X), which correlates with the high number of transposases, integrases, and prophage sequences annotated in each plasmid. Proteins in plasmids pPSLF89-1, pPSLF89-3, and pPSLF89-4 are more diverse in COGs, such as regulation of transcription and translation (K and L in pPSLF89-1, J and K in pPSLF89-3, and L in pPSLF89-4). In lower proportions, proteins related to transport and metabolism of carbohydrates (G) and coenzymes (H) were identified in pPSLF89-3; amino acids (E), nucleotides (F), and lipids (I) were identified in pPSLF89-1; and coenzymes (H) and energy production and conversion category (C) in pPSLF89-4. The pPSLF89-4 plasmid was the only one carrying proteins related to COG U (intracellular trafficking, secretion, and vesicular transport), and correspond to proteins related to type IV secretion apparatus, a plasmid conjugation system. In addition, T-category-related proteins annotated as part as a two-component system and a protein related to peptidoglycan modification or biosynthesis (M) were found exclusively in pPSLF89-1. Putative virulence factors were predicted in pPSLF89-1, pPSLF89-3, and pPSLF89-4 plasmids, but were not categorized in COG, as most of them were unknown (S), or were not determined (N.D. category). The predicted virulence factors with assigned COGs were categorized in I and K (in pPSLF89-1), O (in pPSLF89-3), and in H (in pPSLF89-4). Proteins relevant for plasmid replication and maintenance are grouped in the K, L, and V categories that correspond to proteins related to toxin–antitoxin (TA)-systems, the V COG also includes the endonucleases and restriction enzymes, and D and N COGs are related to plasmid segregation. Plasmid replication proteins were predicted in pPSLF89-4 and pPSLF89-1 plasmids and belong to the N category, or could not be categorized (S or N.D.).

### 2.3. Annotation and Sequence Analysis of P. salmonis LF-89 Plasmids

**Figure 4:**
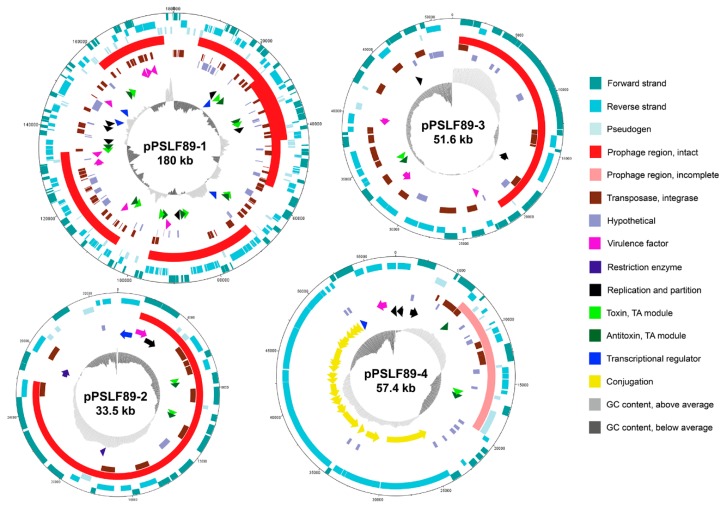
Circular genetic map of *P. salmonis* LF-89 plasmids. Plasmids sequenced by Illumina were annotated with GenDB Metanor using UniprotKB and nr, ProtFun 2.0, SMART, and VFDB. Prophage regions were predicted using PHASTER, and type II toxin–antitoxin (TA) modules with TAfinder. The inner circle represents GC content, plotted as the deviation from the average GC content of the sequence. Genes with predicted functions were grouped into categories (shown in the right panel), and the arrow head indicates the direction of the genes of interest. Genes were assigned to the following categories: prophage region (intact or incomplete); transposase, integrase; hypothetical; virulence factor; nutrient transport and metabolism; restriction enzyme; replication and partition; TA module; and transcriptional regulator and conjugation. Plasmid name and size is shown with black letters in each plasmid.

Besides the size and migration pattern of the LF-89 plasmids, other features were studied in the predicted plasmid sequences in order to investigate their nature and putative functions in *P. salmonis*. Based on the annotation, circular representations of each plasmid were created, as shown in Figure 4. Annotation of LF-89 plasmids revealed the presence of a total of 403 open reading frames (ORFs) that were annotated and sorted into 10 categories: pseudogenes; prophage regions; transposases, integrases; hypothetical proteins; virulence factors; nutrient transport and metabolism; restriction enzymes; TA-systems; replication and partition; transcriptional regulators; and conjugation system. All plasmids CDS predictions are listed in Supplementary Table S2.

#### 2.3.1. Replication and Plasmid Stability Elements

In pPSLF89-1 plasmid, six CDS were annotated as related to plasmid replication or partitioning (Figure 3, Supplementary Table S2). Three of them (PSLF89_RS33890, PSLF89_RS34705, and PSLF89_RS34710) contained the partitioning systems-related domains ParB/RepB/Spo0J or CobQ/CobB/MinD/ParA; one was a putative ATPase involved in DNA repair or chromosome segregation (PSLF89_RS34330), and the two others were described as replication proteins (PSLF89_RS34010 and PSLF89_RS34650). These CDS were unique to *P. salmonis* strains, as observed by nucleotide sequence similarity after a BLAST search. When search and alignment parameters were less strict, the PSLF89_RS34650 sequence was identified as putative *repB* gene similarity to other bacterial plasmid encoded genes such as the *Acinetobacter* sp. plasmid p1_010005, plasmid pRW1, and pABIR (41.5% identity, 62% coverage). The other pPSLF89-1 putative replication protein (PSLF89_RS34010) contains a DNA-binding domain protein that binds to plasmid replication regions. As RepB has not been reported to replicate bacterial plasmids on its own, the replication mechanism of pPSLF89-1 could not be inferred. No *rep* genes were found in any other LF-89 plasmid.

In pPSLF89-4, another type of replication protein, TrfA (locus tag PSLF89_RS35445), was identified along with one CDS with the protein domain ParB/RepB/Spo0J (PSLF89_RS35425) and one with the CobQ/CobB/MinD/ParA (PSLF89_RS35430) protein domain. The replication initiator *trfA* gene does not have a significant identity sequence with any other known plasmid initiator gene. Two CDS with putative partitioning protein domains were found in pPSLF89-2 (PSLF89_RS34925 and PSLF89_RS34930) and pPSLF89-3 (PSLF89_RS35230 and PSLF89_RS35395), but no putative replication proteins. No origin of replication sequences or iterons were identified in either of the LF-89 plasmids. *oriV* and *oriC* sequences from the Genbank plasmid database were compared with LF-89 plasmids with no positive results. In addition to the partitioning systems, several toxin-antitoxin proteins were found as TA-modules in all *P. salmonis* LF-89 plasmids (Supplementary Table S3). Interestingly, pPSLF89-4 encodes a putative type II toxin-antitoxin system Phd/YefM family antitoxin (PSLF89_RS35470), but its cognate toxin (PSLF89_RS35475) is interrupted by an internal stop codon. This suggests that this antitoxin is a pseudogene, and, therefore, is not functional.

#### 2.3.2. DNA Mobilization and Gene Transfer in Plasmids

Proteins annotated as part of the insertion sequences were found in all plasmids, but the larger plasmid pPSLF89-1 encodes a higher amount and greater diversity of transposases. The identified proteins were similar to transposases encoded by insertion sequence elements (see Supplementary Table S2). In all plasmids, IS30 family transposases were also predicted as integrases, but additionally, from one to seven integrases were also observed in the plasmids. The amount of integrase and transposase proteins annotated in each plasmid corresponds to 0.45 elements per kilobase (element/kb) in pPSLF89-1, 0.33 elements/kb in pPSLF89-2, 0.41 elements/kb in pPSLF89-3 and 0.104 elements/kb in pPSLF89-4. According to these values, *P. salmonis* plasmids possess high densities of IS elements when compared to other bacterial pathogens [38].

Other elements found in *P. salmonis* plasmids could potentially mobilize bigger segments of DNA than the ones mentioned above. At 57.4 kb in length, pPSLF89-4 comprises a 20 kb putative type IV secretion system (T4SS) region involved in conjugation. This region includes a Tra system with 20 genes; 18 of them annotated as *tra*- or *trb*-like genes (*traALEKBVC*–*trbI*- *traWU*-*trbC*-*traNF*-*trbB*-*traHGTD*), and two of them located between *traDT* and *traTG* were identified as hypothetical genes with unknown functions (Figure 4). In addition, a putative TraI relaxase, a mobilization protein from the MOBF family of relaxases [39], was identified 6.9 kb upstream of this region. The mosaic composition of the pPSLF89-4 conjugation system, although similar to Ti plasmid from *Agrobacterium tumefaciens* in terms of gene composition [40], shares amino acid sequence similarity with the T4SS from *Legionella*, as seen by BLASTp search. The gene organization of *A. tumefaciens* Ti plasmids differs from the *P. salmonis* pPSLF89-4, since in the former, the *tra* genes are two different clusters organized as divergently expressed operons and *trb* genes are organized in a single operon [40,41], while in the latter plasmid, both systems are intercalated. *tra* genes from *A. tumefaciens* Ti plasmids are also related to IncP and IncQ plasmids, but in *P. salmonis*, the conjugative transfer pilus assembly protein TraK is described as part of the IncF plasmids, which suggests both plasmids belong to different incompatibility groups.

#### 2.3.3. Prophage Sequences and Prophage Induction in *P. salmonis* LF-89

The size of the prophage regions found in pPSLF89-1, pPSLF89-2, and pPSLF89-3 plasmids (Table 2) explains the high number of proteins categorized in COG X, and low variety and proportion of other COGs (Figure 3), especially in pPSLF89-2, where the prophage region comprises 73% of the plasmid sequence. Interestingly, nearly 80% of the pPSLF89-1 plasmid genes corresponded to prophage sequences, arranged in five complete prophage regions (i.e., regions that contain all the necessary proteins to generate viral progeny), and with prophage regions 1 and 2 partially overlapping. The viral proteins corresponded to dsDNA-type phages, non-enveloped with a head–tail structure, of the order Caudovirales. The prophage regions of pPSLF89-1 also encode non-phage-related proteins, most of them with unknown functions or transposases, but also genes of interest such as putative virulence factors and TA-systems, located in the plasmid skeleton and in prophage regions (Figure 4). Plasmid-maintenance-related proteins are encoded inside and outside prophage regions. For example, in regions 1–3, the putative partitioning proteins were found (Figure 4).

In pPSLF89-2—the smallest plasmid found in *P. salmonis* LF-89—most of its 33.5 kb sequence corresponded to a dsDNA prophage from a similar lineage to those observed in pPSLF89-1 (Table 2). The plasmid encodes 37 proteins and 31 of them are viral. No attachment site was identified in this prophage region, although it was classified as an intact prophage. This prophage region carries the partitioning genes and both TA-modules (Figure 4). Of the two genes predicted as restriction enzymes, one was found in the plasmid skeleton and the other, in the prophage region. Similarly, pPSLF89-3 encodes one intact 20.6 kb prophage that comprises almost 40% of the plasmid sequence and no attachment site was found. The prophage region carries structural and mobility viral proteins, but also, the putative chromosome partitioning protein (Figure 4). Proteins with unknown function were also encoded inside and outside the prophage regions, as well as 19 transposases and integrases (Figure 4). The only plasmid predicted to contain one incomplete prophage in the LF-89 strain is pPSLF89-4, the conjugative plasmid (Table 2). The incomplete prophage does not possess the structural proteins required to form a virion (capsid, tail, and portal, among others) and neither lysis proteins, proteases, nor the attachment site were identified. Finally, a complete TA-system is encoded in this prophage, with nine transposases and integrases sequences (orange rectangles, Figure 4).

**Figure 5:**
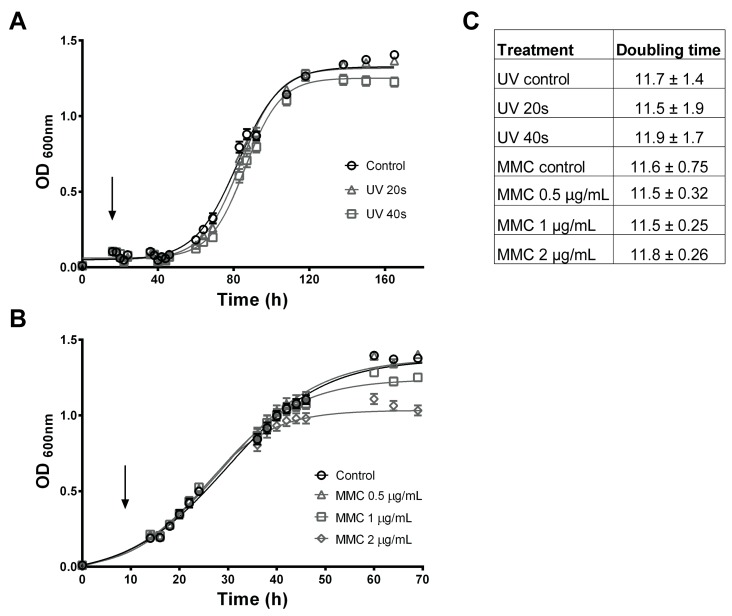
Prophage induction assays in *P. salmonis* LF-89. Prophage induction was evaluated by bacterial growth (OD_600_ measurements over time) of treated and un-treated *P. salmonis* LF-89 cultures. Averages of six independent replicates with their corresponding standard deviations are shown. Black arrows indicate the treatment administration time. (**A**) Growth curves profiles observed in bacteria exposed to UV germicidal light for 40 seconds (UV 40s, white squares), 20 seconds (UV 20s, white triangles), and control un-exposed cultures (control, white circles). (**B**) Growth curves profiles from bacteria treated with 0.5 µg/mL of MMC (white triangles), 1 µg/mL of MMC (white squares), 2 µg/mL of MMC (white diamonds), and untreated cells as control (white circles). (**C**) Doubling time of each growth curve calculated from the exponential growth phase of curves depicted in (**A**,**B**). The numbers of doubling time indicate increase in optical density per hour.

Although all prophage regions found in LF-89 corresponded to the same dsDNA (no RNA stage, Caudovirales type of virus), none of them showed high similarity with other known bacteriophages, suggesting that LF-89 prophages have a combination of viral proteins from different phage species and unknown prophages. In an attempt to stimulate prophage induction, exponentially growing bacterial cells were exposed to the cytotoxic antibiotic mitomycin C (MMC) and to ultraviolet germicidal light (UV). As the induction protocols were not described for *P. salmonis*, a range of MMC concentrations and UV exposure time were first evaluated. After the incubation with both treatments, no significant decrease in cell density in the exponential or stationary growth phases, or in replication capacity (as seen by the doubling time calculation) was observed compared to the control untreated cultures (Figure 5). The decrease in growth capacity observed with the higher MMC concentration is related to the compound toxicity, as phage-induced cell lysis is usually observed at concentrations lower than 1 µg/mL. These results are indicative that no cell lysis caused by phage release occurred, and consequently, the prophage regions contained in the *P. salmonis* genome are not inducible, at least by these methods.

#### 2.3.4. Plasmid Virulence Factors 

Proteins containing functional domains associated with nutrient acquisition, adhesion, drug transport, bacterial replication inside the host, and host-cell cytotoxicity were identified in *P. salmonis* plasmids. A total of 14 proteins fulfilling these criteria were further analyzed. Two of them corresponded to transcriptional regulators identified in pPSLF89-1, a CsrA family protein carbon storage regulator (PSLF89_RS34715), responsible for bacterial metabolic shifts and stationary-stage phenotypes including virulence in other pathogens such as *L. pneumophila* [42], and a Crp/Fnr family transcriptional regulator (PSLF89_RS34760), which responds to a broad spectrum of signals such as temperature, carbon monoxide, anoxia, cAMP levels, redox state, or oxidative and nitrosative stress [43]. These proteins could be related to bacterial survival in different environments, but have not been commonly described as bacterial virulence factors.

On the other hand, a total of 13 ORFs were predicted as putative virulence factors (VF) in the plasmids (Supplementary Table S2), six of them were predicted by sequence similarity searches against the VFDB. Thus, pPSLF89-1 plasmid harbors a PLD-like domain protein (PSLF89_RS34605) related to Ymt murine toxin from *Yersinia pestis* [44] and two pentapeptide repeat-containing protein PipB2 (PSLF89_RS34855 and PSLF89_RS34870) involved in the intracellular replication of bacteria enclosed in cytoplasmic vesicles, such as *Salmonella enterica* [37]. Another pentapeptide repeat-containing protein (locus tag PSLF89_RS35360) similar to *Salmonella enterica* serovar Paratyphi PipB2 was encoded in pPSLF89-3. In pPSLF89-2, the only predicted VF (PSLF89_RS34925) is similar to *Campylobacter fetus flhG* ATPase, which is part of the flagella apparatus. It is tempting to speculate that this CDS could be part of the incomplete flagella apparatus predicted in *P. salmonis* LF-89 chromosome, which could be used as a secretory complex [45]. However, this CDS is also similar to partitioning protein ParA and it is the only CDS identified as a ParA family protein in pPSLF89-2, so it is more likely to be an ATPase involved in the partitioning function rather than in virulence. Thus, this CDS was not considered to be a VF. pPSLF89-4 also encodes only one VF (PSLF89_RS35745), predicted as a glutamate-1-semialdehyde-2,1-aminomutase similar to *Haemophilus somnus* hemL. In *H. somnus*, this gene participates in Heme biosynthesis [46] as part of an iron uptake mechanism. 

The other seven plasmid-encoded VFs either contained eukaryotic protein domains, or their function was described as relevant for the infection process in other pathogenic bacteria. In pPSLF89-1, five CDS were annotated as putative VFs. PSLF89_RS34355 encodes a Toll/interleukin-1 receptor (TIR) domain protein, and bacterial TIR-containing proteins have been described to contribute in host innate immune system evasion [47]. A eukaryotic-domain- containing protein (PSLF89_RS34625) belongs to the Fic family, which, in pathogenic bacteria, are secreted effector proteins that mediate post-translational modifications of host-cell proteins [48]. PepO endopeptidase (PSLF89_RS34880) was described in *Streptococcus pneumoniae* as a plasminogen- and fibronectin-binding protein that participates in invasion and evasion of host immunity [49]. The remaining VFs were not described in other bacteria, but their annotated functional domains enabled them to be classified as VFs. PSLF89_RS34755 encodes a multidrug ABC transporter permease, and a spore coat protein (PSLF89_RS34305), which was predicted as a surface protein that could be related to cell protection. pPSLF89-3 encoded VFs contain eukaryotic-domains, and have a general function description. One is a Ras family protein (PSLF89_RS35310), responsible for animal cell proliferation among other functions [50,51], and the other is a tetratricopeptide repeat (TPR) family protein (PSLF89_RS35270), which participates in protein–protein interactions and has been described to contribute in bacterial virulence [52,53].

### 2.4. Plasmid Genes Predicted as Virulence Factors Were Overexpressed during Infection of Salmon Macrophages

**Figure 6:**
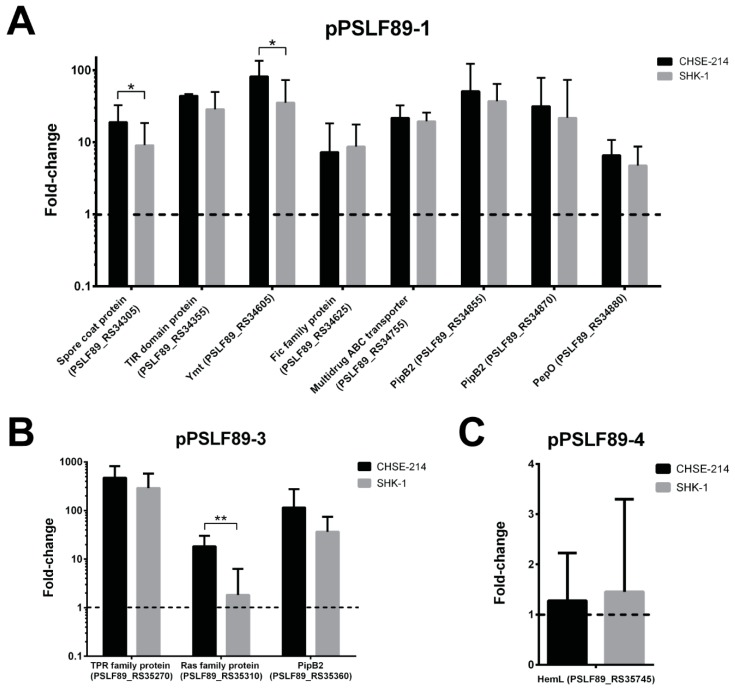
Expression levels of *P. salmonis* LF-89 plasmid virulence factors during infection in salmon-derived cell lines. RT-qPCR of predicted plasmid-encoded virulence factors in the LF-89 strain during infection in CHSE-214 and SHK-1 cell lines. Expression levels are shown as fold-change expression relative to *glyA* and *pykA P. salmonis* housekeeping genes. The dotted black line indicates the control levels. Statistical differences between virulence factors (VFs) expression in cell lines were calculated for each case separately with a paired t-test (* *p* < 0.05, ** *p* < 0.01). Relative expressions of VFs encoded in pPSLF89-1 are shown in (**A**), pPSLF89-3 in (**B**), and pPSLF89-4 in (**C**).

The expression levels of the 12 genes predicted as plasmid VFs (five with homology to VFDB and seven with prediction based on functional domains) were evaluated during in vitro infection of two salmon-derived cell lines, the Chinook salmon embryo cell line CHSE-214 and the macrophage-like SHK-1 cell line from Atlantic salmon. Expression levels were normalized by housekeeping *P. salmonis* genes and were quantified by RT-qPCR. We found that all predicted virulence factors in plasmids pPSLF89-1 and pPSLF89-3 were overexpressed in both cell lines (Figure 6). In general, expression levels of these genes were similar in CHSE-214 and SHK-1 cells, with the exception of two pPSLF89-1 putative virulence factors—the spore coat protein gene (PSLF89_RS34305) and Ymt toxin (PSLF89_RS34355))—and one pPSLF89-3 gene (PSLF89_RS35310) that showed significantly higher expression levels in CHSE-214 cell line (*p* < 0.05). In both infected cell lines, the *hemL* gene (PSLF89_RS35745), the only pPSLF89-4-predicted VF, did not change its expression levels during infection (Figure 6).

### 2.5. Plasmid Virulence Factors are Shared and Highly Conserved among Other P. salmonis Strains

Up to February 2018, twenty *P. salmonis* strains have been sequenced, annotated, and deposited in the NCBI database. Annotation results reported plasmid replicons in each of the sequenced strains, ranging from two to eight plasmids per strain (Table 3). The wide distribution of plasmid sequences among all known *P. salmonis* strains suggests a role in the bacterial survival or maintenance in its biological niche.

Considering that we have previously shown that LF-89 plasmids carry several putative virulence factors that are overexpressed during infection, we used BLAST to search for individual virulence factors sequences in all *P. salmonis* genomes (Table 3 and Supplementary Table S6). The BLAST parameters used in this search were strict both in terms of coverage and in sequence similarity due to the presence of highly similar coding sequences among *P. salmonis* strains. As shown in Table 3 and Supplementary Table S6, all LF-89 plasmid-encoded virulence factors were identified in other *P. salmonis* strains. Some virulence factors such as the TIR domain protein and Ymt (pPSLF89-1) were identified in all *P. salmonis* strains, while others were found in less than 50% of the strains, such as the TPR family protein gene (pPSLF89-1) that was found in eight of the 19 strains, or the Ras family protein gene (in pPSLF89-3), which was identified only in seven strains (Supplementary Table S6). Interestingly, orthologues of LF-89 plasmid VFs were identified in the plasmids, in the chromosome, or in both types of replicons in the other *P. salmonis* strains. Of all the 12 predicted LF-89 VFs, eight were identified only in bacterial plasmids and four in plasmids and chromosomes. The TIR domain protein, the multidrug permease, the Ymt toxin, and the TPR family proteins were found in the plasmids and chromosomes of some strains; the latter was duplicated in three strains, as seen by the presence of two copies of the gene, one in plasmids and one in the chromosome. It is worth noticing that pPSLF89-1 VFs were more represented than the VFs encoded in pPSLF89-3 and pPSLF89-4, as seen by a higher percent of strains carrying them.

The majority of virulence factors in the LF-89 strain are encoded in the pPSLF89-1 plasmid, and three of them were found exclusively in strains of genogroup LF89-like (Table 3). Furthermore, most of the pPSLF89-1 virulence factors were found in other plasmids from genogroup LF89-like strains. The pPSLF89-1-encoded VFs could be encoded either in a plasmid or in the chromosome in genogroup EM90-like strains. Virulence factor genes from pPSLF89-3 and pPSLF89-4 plasmids were not identified in the genome of genogroup EM90-like, but only in genogroup LF89-like strains (Table 3). Interestingly, the tetratricopeptide-repeat (TPR) family protein gene in pPSLF89-3 was identified both in plasmids and in the chromosome of some genogroup LF89-like strains. It was the only virulence factor with a chromosomal paralog in genogroup LF89-like.

## 3. Discussion

In this study, we identified and characterized four *P. salmonis* plasmids and their gene content, including virulence factors genes. Based on their nucleotide sequence, specific probes were designed that allowed plasmid visualization and discrimination.

Ontology analysis revealed the presence of typical plasmid functions, such as replication and partition, suggested by plasmid-encoded proteins with ParA, ParB, MinD, CobB, CobQ, RepB, or Spo0j domains. The presence of partitioning mechanisms is a common feature in low-copy plasmids [54], a feature that was also observed in all LF-89 plasmids. Although these functions were predicted from the annotation, no replication initiator proteins such as RepA were identified in *P. salmonis* pPSLF89-1, pPSLF89-2, and pPSLF89-3 plasmids, and consequently, no replication mechanism could be inferred. The exception was the pPSLF89-4 plasmid, which encodes a TrfA protein required for initiation of plasmid DNA replication in a DnaA-dependent manner [11]. In addition, this conjugative plasmid was the only one that encoded a mobilization protein (TraI), necessary for the transmissibility of the plasmid via the T4SS. It should also be noted that plasmid replication genes and their associated incompatibility groups could not be identified using PlasmidFinder [55].

Considering that of *oriV* and *oriC* replication sites could not be predicted with standard methods in *P. salmonis* plasmids and the fact that that they are low-copy-plasmids (1–2 per cell), we hypothesized that *P. salmonis* plasmids could be passively replicated along with the chromosome. However, plasmid replication was not visualized or otherwise tested, so further experiments would be required to elucidate the replication mechanism of *P. salmonis* plasmids.

Another widely described plasmid maintenance system is the toxin–antitoxin (TA) module, which contributes to the heritability of the plasmids to the daughter cells. The TA systems consist of a potentially harmful module, the toxin, which is counteracted by the antitoxin module, an unstable protective component [44]. Despite the fact that TA-systems could be underestimated, twelve different protein–protein TA modules were identified in LF-89 plasmids; nine of them were encoded in prophage regions (Figure 4). Integrated phage sequences, known as prophages, were predicted in all *P. salmonis* LF-89 plasmids. Five intact prophages were identified in pPSLF89-1 plasmid, one in pPSLF89-2 and pPSLF89-3 plasmids, and one incomplete prophage region in pPSLF89-4 (Table 2); however, no bacterial lysis was observed under culture-conditions. Moreover, the prophage sequences could not be induced with either mitomicyn C or UV light (Figure 5), suggesting that the predicted prophages are unable to form progeny, at least under our growth conditions. Only one study has reported the presence of *P. salmonis* phages by microscopy observations [56], although the nature and classification of those phages remains unknown.

Although in this work functional analysis of the TA systems was not conducted, previous studies have shown that *P. salmonis* possess a functional TA-module, as proved by heterologous expression in *Escherichia coli* [57]. The co-occurrence of partitioning proteins, TA-systems and prophage regions suggest an evolutionary function and possibly, an interaction between bacterial cells and the phages that are parasitic to the bacteria. Strong interactions between bacterial cells and phage genomes can create permanent prophages that stay in the bacterial genome as episomes or even plasmids, which replicate and propagate along with the bacteria [46]. Prophages could be unstable elements and disrupt important bacterial functions; consequently, many important genomic regions lack this type of sequence, as well as other mobile elements such as transposons [46]. This was observed in pPSLF89-4, where all predicted transposases and integrases were located outside the T4SS region (Figure 4).

It is interesting to note that no CRISPR-Cas or DISARM immunity systems were identified in the *P. salmonis* LF-89 genome, which could partially explain the high proportion of phage-related sequences in the bacterial genome. However, the existence of immunity against phages in *P. salmonis* cannot be discarded since other anti-phage defense systems have been reported in microbial genomes, and new systems are being identified [58]. For example, the pPSLF89-2 plasmid encodes two putative restriction enzymes. One of them is described as a type I restriction modification DNA specificity domain protein, and the other, as a restriction endonuclease subunit S protein. Although incomplete, this could be part of a type I restriction–modification system [59] (or related system) that in the LF-89 genome is encoded in a plasmid composed primarily of an intact phage. The restriction/modification systems are considered an ancient form of a genetic immunity system, closely associated with group immunity—a strategy used by prokaryotes for group identification, which protects identity and harms the non-identical [46]. Described as part of a co-evolutionary dependency, it is thought that persisting genetic parasites such as prophages can encode restriction modification enzymes and use them as addiction modules to ensure their maintenance and stability inside their host [46]. Thus, the presence of prophages in the LF-89 genome could indicate an ongoing inter-relationship between the first *P. salmonis* isolate and its environment that has prevailed in laboratory conditions.

Due to the highly variable nucleotide sequence of Caudovirales phages [60], and the unknown nature of the predicted phages species, it was not possible to determine if the different prophage regions correspond to multiple insertion events of the same phage, or to different phages. The high prevalence of prophages, transposases, and integrases sequences in *P. salmonis* LF-89 plasmids suggest that DNA exchange could occur between different genetic elements (chromosome–chromosome, chromosome–plasmids, and/or plasmid/plasmid DNA exchange events), which, in addition to the presence of a conjugative plasmid supports the notion that the *P. salmonis* genome is plastic and possesses a high capacity to exchange and acquire exogenous DNA. This is supported by the evidence that *P. salmonis* has an open-state pan genome, and is exemplified by the different structural configurations for the six identified copies of the ribosomal operon in the *P. salmonis* genome, found in comparative genome analyses from 19 strains [6], suggesting translocation throughout the *P. salmonis* genetic material. In *P. salmonis* plasmids, several prophage genes were classified as unknown or had no relation with the phage life-cycle. For example, several TA-systems, replication and partition proteins, transcriptional regulators, and virulence factors were found in these regions (Figure 4). This could be related to the role of *P. salmonis* plasmids (and the plasmid-related prophages) in the bacterial life-style and pathogenicity.

Interestingly, *P. salmonis* plasmids were found to be highly novel, as observed by low similarity to the NCBI plasmid database. Therefore, we speculated that the novelty of the plasmid sequence, added to the high content of hypothetical or unknown proteins encoded in *P. salmonis* plasmids (Figure 4), could be related to the plasmids’ function in the life cycle of this bacterium. *P. salmonis* plasmids could contribute to bacterial fitness by supporting its growth both inside and outside the host. Plasmid contribution to the intracellular lifestyle of the bacteria is suggested by the numerous virulence factors that they harbor. Some of them are described in other bacteria as toxins or proteins that allow bacterial invasion and propagation, such as endopeptidase O (PepO in *Streptococcus pneumoniae* [49]). Interestingly, two transcriptional regulators encoded in pPSLF89-1, Crp/Fnr, and CsrA have been described as playing a role in bacterial adaptation to stress and virulence [42,43]. Although CsrA was first described as a chromosomal regulator of metabolic pathways in *E. coli* [61], it has also been found in mobile elements in bacteria that lacks a chromosomal copy of this regulator, such as *Sinorhizobium meliloti* [62], and in bacteria that possess a chromosomal *csrA*, such as *L. pneumophila* Corby [63]. In the latter, the plasmidial CsrA was speculated to be involved in the regulation of other plasmidial genes such as a conjugation-type secretion system [63]. This could be the case for *P. salmonis*, as it has both a chromosomal and plasmidial *csrA* gene.

A total of 12 genes, distributed in pPSLF89-1, pPSLF89-3, and pPSLF89-4 plasmids, were predicted as virulence factors. Plasmid pPSLF89-1 harbors the majority of the VFs, although they were also found in pPSLF89-3 and pPSLF89-4. Since virulence-associated genes specifically expressed during host infection are candidate virulence determinants, we used two cell-culture infection models, one from embryonic origin (CHSE-214 cells from Chinook salmon) and other fibroblast-like cells with immune cell characteristics (SHK-1 cells from Atlantic salmon), to maximize the probability of observing the expression of VF genes. The expression during the infection process is an important pre-requisite to characterize a VF, and we observed an increased expression of all pPSLF89-1 and pPSLF98-3 predicted VFs, which strongly suggests a role of these genes during *P. salmonis* pathogenesis. Although the predicted pPSLF89-4 VF did not overexpress in the CHSE-214 and SHK-1 cells, we could not rule out that they could function as VFs in another context. It is also noteworthy that the conjugative plasmid pPSLF89-4 could spread this VF, along with other genes, across the *P. salmonis* population. These functions could be necessary for the bacterium pathogenicity as it moves from the marine environment to the host, or to survive nutrient scarcity.

Another characteristic that supports the relevance of these predicted plasmidial VFs is the conservation of the sequences in other *P. salmonis* strains. Some LF-89 predicted virulence factors were found in all 19 sequenced and annotated *P. salmonis* strains’ genomes (such as two toxins and a TIR domain protein encoded in pPSLF89-1 plasmid), and some in less than half of the sequenced strains (such as an iron transporter from pPSLF89-1, a Ras family protein, and a TPR family protein from pPSLF89-3 plasmid). Most of the LF-89 virulence factors were identified in the closely related LF-89-like genogroup. The case of PipB2 is noteworthy, which in *Salmonella* is related to the formation of the replication vacuole [37], and is present in four copies in the LF-89 genome: two in the pPSLF89-1 plasmid, one in the pPSLF89-3 plasmid, and one in the chromosome. Ortholog genes were identified in 12 strains of genogroup LF89-like with the same distribution (three in plasmids and one chromosomal copy), which suggest a high conservation among *P. salmonis* strains. *P. salmonis* strains harboring virulence factors in plasmids that are conserved among strains may lead to recombination and selection of epidemiologically important plasmids by transferring virulence determinants and increasing virulence in the host. Moreover, shared genes between chromosomes and plasmids of some *P. salmonis* strains suggest that gene exchange events between them have occurred during the evolutionary history of the *P. salmonis* group. It has been hypothesized before that recombination between IS copies may lead to genome rearrangements by homologous recombination [38], which could be the case for *P. salmonis* diversity in the location of the observed VF. The ability to preserve adaptive genes (in this case, VFs) on chromosomes or plasmids has been regarded as a survival strategy of bacteria to face frequent changes of environmental conditions [64].

## 4. Materials and Methods

### 4.1. Bacterial Strains and Growth Conditions

The *P. salmonis* LF-89 strain (ATCC VR-1361) used in this study was obtained from the American Type Culture Collection (ATCC).

The bacteria were routinely maintained by sub culturing in liquid broth (AUSTRAL-SRS [65]) with agitation (180 rpm) at 18 °C. Each subculture was confirmed as *P. salmonis* by Gram stain and PCR-RFLP assays [66].

### 4.2. Purification of P. salmonis Plasmids

Isolation of *P. salmonis* plasmids was carried out by a column-based method with the Qiagen^®^ Plasmid Midi Kit (Qiagen Group, Germany), a commercial kit for purification of ultrapure plasmid DNA. Prior to DNA extraction, the cultured bacteria were centrifuged for 15 min at 6000 × *g* and the cell pellet washed twice with PBS (phosphate-buffered saline, 137 mM NaCl, 2.7 mM KCl, 10 mM Na_2_HPO_4_, and 1.8 mM KH_2_PO_4_). The purified plasmids were loaded into a 0.8% agarose gel and electrophoresis was performed to evaluate the plasmid isolation method.

### 4.3. Plasmid Sequencing and Annotation

*P. salmonis* LF-89 plasmids were sequenced by Illumina GAIIx using a shotgun library and a total of 70,531,338 paired-end reads of 101 bp was generated (2064× raw coverage). The resulting sequences were compared with the PacBio sequences already published by Pulgar et al. [5]. Putative coding sequences (CDS) were predicted using Glimmer 3.02 and GeneMarkS. Functional annotation of CDS was performed using Metanor of GenDB with UniprotKB/Swiss-Prot, KEGG, and nr. Protein domains were identified using InterPRO-scan against the Inter-PRO database collection. Virulence factors were identified using the Virulence Factor Data Base (VFDB). Furthermore, ProtFun 2.0 and SMART online tools were used to predict unknown virulence factors based on the presence of secretion signals, functional or eukaryotic protein domains in plasmid-encoded hypothetical proteins or proteins with unknown function. Prophage regions were identified and annotated in the bacterial chromosome (NZ_CP011849.2), or the plasmid sequences (CP011850.1, CP011851.1, CP011852.1, and CP013669.1) using the online tool PHASTER with standard parameters [67,68]. Finally, type II toxin-antitoxin systems were searched using the online tool TAfinder [69]. Plasmid graphical representations were created using DNAPlotter [70].

Predicted CDS were assigned to a COG category using the Cluster of Orthologous Groups (COG) eggnog database [71]. COGs were manually curated if proteins were classified into two or more COGs, or no COG was assigned (grouped as non-determined category or N.D.)

### 4.4. Design and Synthesis of Digoxigenin-Labeled Oligonucleotides

Dig-labeled probes were designed to hybridize with an exclusive and specific zone in each plasmid (pPSLF89-1, pPSLF89-2, pPSLF89-3, and pPSLF89-4). In order to find specific probes for each replicon, a bioinformatics approach was developed using the predicted CDS. Starting with a CD-HIT-EST (95% identity), CDS that formed clusters with others were discarded. Then, a bidirectional megablast was performed using the remaining CDS with a high e-value (1e–2). Candidate probes were selected from CDS with a unique hit (a hit against itself). Then, the longest probes were selected.

Purified bacterial plasmids were used as templates for the PCR with the primers listed in Supplementary Table S4. The PCR reaction was performed with a Taq polymerase (Thermo Fisher Scientific) according to the manufacturer instructions, using Taq Buffer with (NH_4_)_2_SO_4_, 2.5 mM MgCl_2_, and supplemented with 5% DMSO. The PCR product was loaded into a 0.8% agarose gel and the bands corresponding to the probes were excised and purified using an E.Z.N.A. ^®^ Gel Extraction Kit (Omega Biotek, Norcross, GA, USA). The purified probes were submitted to a second PCR incorporating the Dig-labeled nucleotides, using DIG DNA Labeling Mix (Roche Diagnostics GmbH, Germany) according to the manufacturer’s instructions. The PCR products were subsequently purified from a 0.8% gel after electrophoresis, and the purified probes were quantified using a Nanophotometer (LabelGuard™ Microliter, dsDNA mode, Nanophotometer™ IMPLEN 2.1, Western Village, CA, USA).

### 4.5. Southern Blot Analysis

Purified DIG-labeled probes were first tested in a dot blot analysis for labeling efficiency as recommended by the manufacturer, using two-fold dilutions for each probe spotted directly on a membrane and visualized with standard DIG detection procedures (2008. DIG Application Manual for filter hybridization. Roche Diagnostics GmbH, Mannheim, Germany).

*P. salmonis* LF-89 were separated in a 0.8% agarose gel by an electrophoresis in TBE buffer (89 mM Tris, 89 mM boric acid, and 2 mM EDTA, pH 8.3) at 40 mV for 5 hours. After staining and photographing the gel, the DNA was transferred and cross-linked to positively charged nylon membranes, hybridized against each DIG-labeled probe and revealed accordingly to the manufacturer’s instructions. Briefly, the cross-linked membranes were washed with 2 × saline-sodium citrate (SSC) buffer (300 mM NaCl and 30 mM trisodium citrate, pH 7), dH2O and then incubated with DIG Easy Hyb buffer (Roche) at 42 °C. DIG-labeled probes were denatured at 95 °C for 5 min, and then co-incubated with the membranes in DIG Easy Hyb buffer (Roche) at 42 °C over-night. After incubation, membranes were washed twice in SSC 0,1X + SDS 0.1% for 10 min at 68 °C and maleic acid buffer (100 mM maleic acid and 150 mM NaCl, pH 7.5) at room temperature, and then blocked with blocking reagent (100 mM maleic acid, 150 mM NaCl, 5% skim milk, pH 7.5) for 30 min. Membranes with DIG-labeled probes were detected with anti-digoxigenin-AP (100 mM NaCl, 100 mM Tris, 5 mM MgCl_2_, pH 9.5), NBT and BCIP (Sigma-Aldrich). The dyed membranes were washed with dH_2_O and photographed for analysis.

### 4.6. Plasmid Copy Number

*P. salmonis* LF-89 plasmid copy number was calculated by qPCR as described by Škulj et al. [72]. Briefly, *P. salmonis* LF-89 strain was grown in AUSTRAL-SRS liquid media and aliquots of 1.5 mL were taken in quadruplicate during the exponential growth phase (2 days) and stationary phase (6 days). Aliquots were immediately heated at 95 °C for 10 min and frozen at –20 °C. All samples were thawed and diluted 1/1000 prior to qPCR. The samples were quantified using a Takyon qPCR Kit (Eurogentec) with specific primers (detailed in Supplementary Table S5). The efficiency of the primers for each sample was used to calculate the plasmid copy number, related to the chromosomal copy number, as described previously [72]. Real-time PCR (qPCR) was performed in an AriaMx 1.0 system (Agilent) with the following PCR conditions: 95 °C for 3 min followed by 95 °C for 3 s, 60 °C for 15 s, and 72 °C for 15 s for a total of 40 cycles. Melting curves (1 °C steps between 60 and 95 °C) ensured that a single product was amplified in each reaction. For each sample, the geometric median of the housekeeping genes *glyA* and *pykA* was calculated and used to determine the relative expression levels of the virulence factor genes, using the method described by Pfaffl [73]. Graphical representation and statistical analysis were performed using GraphPad Prism software version 6.01.

### 4.7. Prophage Inductions

Lytic capability of the *P. salmonis* LF-89 predicted prophages was evaluated by induction trials using the intercalating agent mitomycin C (MMC) and Ultraviolet (UV) light, as described before [74], with some modifications. Exponentially growing *P. salmonis* cultures in nutrient broth (AUSTRAL-SRS) were used to inoculate 48-well plates (for MMC induction experiments) and 5 mL cultures (for UV exposure) to an OD_600_ = 0.01. Cultures were incubated at 18 °C for approximately 16 hours until reaching OD_600_ ≈ 0.2. MMC ranging from 0.5 to 2 µg/mL final concentration were added to 48-well plates and the OD600 was monitored using a Tecan microplate reader (NanoQuant™ Infinite^®^ M200 PRO, Thermo Fisher Scientific, Waltham, MA, USA). Five mL cultures were pelleted, suspended in 0.1M MgSO_4_ sterile solution and exposed to 0, 20, or 40 s of germicidal UV light. Treated bacteria were placed in nutrient broth (AUSTRAL-SRS) in 48-well plates, incubated and monitored as for the MMC-treated plate. Bacterial growth curves were plotted and the doubling time of each culture was calculated using the GraphPad Prism software version 6.01.

### 4.8. Cell Cultures

Two salmon-derived cell lines used in this study were obtained from the European Collection of Authenticated Cell Cultures. The epithelial-like embryo cell line CHSE-214 (ECACC 91041114), derived from Chinook salmon *Oncorhynchus tshawitscha*, was grown as recommended in minimum essential medium (MEM) supplemented with 5% FBS (Gibco), at 20 °C in T25 or T75 sterile flasks (Falcon). The macrophage-like cell line SHK-1 (ECACC 97111106) derived from Atlantic salmon (*Salmo salar*) head kidney were routinely grown at 20 °C in T25 or T75 tissue culture flasks with filter, with Leibovitz’s L-15 medium (Gibco) supplemented with 2 mM L-glutamine (Gibco), 40 µM β-mercaptoethanol and 10% FBS (Gibco).

### 4.9. P. salmonis Infection in Cell Cultures

Cell cultures were seeded at 80% confluence in four T75 flasks and incubated over night at 20 °C to attach. Bacteria were grown in liquid broth for four days and inoculated at a multiplicity of infection (MOI) of 100. After 3 days of co-incubation, gentamicin was added to a final concentration of 50 µg/mL, to kill extracellular bacteria. The antibiotic was incubated for 1 hour, washed three times with PBS, and replaced with fresh culture media. Infection was carried out for 12 days until the collection of cell cultures for RNA purification.

### 4.10. Nucleic Acids Purification and Transcripts Quantification 

RNA was purified from the infected CHSE-214 and SHK-1 cell lines with TRIzol (Thermo Fischer Scientific) following the manufacturer’s instructions. RNA was suspended in Ambion^®^ RNAsecure™ (Invitrogen), treated with RQ1 RNase-Free DNase (Promega) according to standard protocols, and visualized in an RNA denaturizing agarose gel. The purified RNA was quantified using a Qubit™ RNA HS Assay kit (Thermo Fischer Scientific), and 2 µg of RNA were used to synthesize cDNA with the M-MLV Reverse Transcriptase using random primers (Promega). Transcripts were quantified using a Takyon qPCR Kit (Eurogentec) with specific primers designed for the predicted *P. salmonis* LF-89 plasmid virulence factors. Real-time PCR (RT-qPCR) was performed in an AriaMx 1.0 system (Agilent), as described above for plasmid copy number calculations.

## 5. Conclusions

Complete nucleotide sequences of four *P. salmonis* plasmids were reported. The genome content of these plasmids suggests the presence of replication-related proteins, partitioning proteins and TA system for plasmid stabilization, and, at least in one of them, self-mobilization through conjugation. Mobilization of genes could also occur by transposases and integrases spread across the four plasmids sequence.

Intact prophage sequences found in three of the four plasmids proved to be un-inducible with MMC and UV, suggesting they could not produce viral particles. *P. salmonis* prophage sequences carry not only viral proteins, but also proteins related to plasmid maintenance and replication and bacterial fitness, which suggests an evolutionary relationship between the prophages and the plasmids that allowed their maintenance over time.

*P. salmonis* LF-89 harbors virulence factors in plasmids that are expressed during infection in salmon cells, and are conserved in other *P. salmonis* sequenced strains. These VFs were identified in other *P. salmonis* strains, encoded in plasmids and/or their chromosome. Thus, it would be of interest to study *P. salmonis* plasmids spreading among field isolates.

## Figures and Tables

**Table 1:** Features of *Piscirickettsia salmonis* LF-89 predicted plasmids.

*P. salmonis* LF-89					
Plasmid	Size (bp)	%GC	Proteins	Genes	Pseudogenes
pPSLF89-1	180,124	38.90	189	233	42
pPSLF89-2	33,516	40.61	37	44	7
pPSLF89-3	51,573	39.06	56	60	4
pPSLF89-4	57,445	37.28	59	66	7

**Table 2:** Prophages encoded in *P. salmonis* LF-89 genome. Prophage regions identified with the PHASTER tool are shown, as well as the size, position inside the plasmid, GC content, and the number of phage species identified based on the protein similarity. All of them correspond to dsDNA viruses with no RNA stage and belong to the order Caudovirales.

Replicon	Prophage Region	Size (kb)	Position in the Replicon	% GC	Percent of Phage Proteins	Percent of Hypothetical Proteins	Number of Phage Species
Chromosome	1 (intact)	38.9	2,079,639–2,118,283	36.9	41.2	58.8	11
pPSLF89-1	1 (intact)	36.4	6546–42,955	37.9	63.3	36.7	9
	2 (intact)	30.8	25,095–55,982	39.1	65.4	34.6	7
	3 (intact)	28.1	68,521–96,644	39.6	83.3	16.7	6
	4 (intact)	28.5	105,452–134,001	38.4	46.4	53.6	3
	5 (intact)	18.2	159,247–177,535	38.2	61.9	38.1	4
pPSLF89-2	1 (intact)	24.5	1476–25,982	40.56	62.9	37.1	14
pPSLF89-3	1 (intact)	20.6	609–21,232	41.2	65.4	34.6	11
pPSLF89-4	1 (incomplete)	12.9	6733–19,700	39.6	52.2	47.8	11

**Table 3:** List of *P. salmonis* LF-89 plasmid-encoded virulence factors identified in other *P. salmonis* sequenced strains. A summary of sequenced and annotated *P. salmonis* strains deposited in the NCBI GeneBank database, the strain name, year of isolation, genogroup, and their assembly code are shown. *P. salmonis* LF-89 plasmids and their VFs, and the location of homologous sequences in other *P. salmonis* strains are indicated. Nucleotidic sequences of VFs were aligned, using the BLAST online tool, against *P. salmonis* sequences deposited in the GenBank database. VF homologues were selected if coverage was > 92% and sequence identity > 85% to LF-89 sequences. Pink boxes indicate the presence of the VF in chromosome or plasmids of *P. salmonis* strains.

	Strain	Year of Isolation	Assembly	Replicons	TIR Domain Protein (PSLF89_RS34355)	Multidrug ABC Transporterpermease (PSLF89_RS34755)	Spore Coat Protein (PSLF89_RS34305)	Ymt Toxin (PSLF89_RS34605)	Fic/DOC Family Protein (PSLF89_RS34625)	PipB2 (PSLF89_RS34855)	Pentapeptiderepeats Family Protein PipB2 (PSLF89_RS34870)	Endopeptidase O (PSLF89_RS34880)	Tetratricopeptide Repeat Family Protein (PSLF89_RS35270)	Ras Family Protein (PSLF89_RS35310)	Pentapeptide Repeats Family Protein PipB2 (PSLF89_RS35360)	HemL Glutamate-1-semialdehyde Aminotransferase (PSLF89_RS35745)
Genogroup LF89-like	LF-89 ATCC VR-1361	1989	GCA_000300295.4	chromosome												
				pPSLF89-1												
				pPSLF89-2												
				pPSLF89-3												
				pPSLF89-4												
	PSCGR01	2010	GCA_001514395.1	chromosome												
				pPsCRG01-1												
				pPsCRG01-2												
				pPsCRG01-3												
				pPSCRG01-4												
	PM22180B	2011	GCA_001932895.1	chromosome												
				p1PS13												
				p2PS13												
				p3PS13												
				p4PS13												
	PM25344B	2011	GCA_001932955.1	chromosome												
				p1PS14												
				p2PS14												
				p3PS14												
				p4PS14												
	PM31429B	2012	GCA_001932915.1	chromosome												
				p1PS12												
				p2PS12												
				p3PS12												
				p4PS12												
	PM32597B1	2012	GCA_000756415.3	chromosome												
				pPSB1-1												
				pPSB1-2												
				pPSB1-3												
				pPSB1-4												
	CGR02	2013	GCA_001534725.1	chromosome												
				pPSCRG02-1												
				pPSCRG02-2												
				pPSCRG02-4												
	AY3800B	2013	GCA_001746795.1	chromosome												
				p1PS10												
				p2PS10												
				p3PS10												
				p4PS10												
	AY3864B	2013	GCA_001932935.1	chromosome												
				p1PS11												
				p2PS11												
				p3PS11												
				p4PS11												
	PM49811B	2014	GCA_001932815.1	chromosome												
				p1PS6												
				p2PS6												
				p3PS6												
				p4PS6												
	PM58386B	2015	GCA_001932835.1	chromosome												
				p1PS7												
				p2PS7												
				p3PS7												
				p4PS7												
	AY6297B	2015	GCA_001932855.1	chromosome												
				p1PS8												
				p2PS8												
				p3PS8												
				p4PS8												
	AY6532B	2015	GCA_001932875.1	chromosome												
				p1PS9												
				p2PS9												
				p3PS9												
				p4PS9												
Genogroup EM90-like	EM-90	1990	GCA_003850185.1	chromosome												
				pPSEM90-1												
				pPSEM90-2												
				pPSEM90-3												
				pPSEM90-4												
				pPSEM90-5												
				pPSEM90-6												
				pPSEM90-7												
				pPSEM90-8												
	PM15972A1	2010	GCA_000756435.3	chromosome												
				pPSA1-1												
				pPSA1-2												
				pPSA1-3												

## Supplementary Materials

The following are available online at https://www.mdpi.com/2076-0817/8/4/269/s1, Table S1: *P. salmonis* plasmid proteins categorized according to Cluster of Orthologous Groups (COG) database. Table S2: *P. salmonis* plasmid complete annotation. Table S3: Prediction of Toxin-antitoxin (TA) modules in *P. salmonis* LF-89 plasmids. Table S4: Specific probes to identify *P. salmonis* LF-89 plasmids by southern blot. Table S5: Primers used for transcript quantification by qPCR. Table S6: List of P. salmonis LF-89 plasmid encoded VFs identified in other *P. salmonis* sequenced strains.
